# Multigenerational inheritance of breathing deficits following perinatal exposure to titanium dioxide nanoparticles in the offspring of mice

**DOI:** 10.1186/s11671-023-03927-0

**Published:** 2024-01-23

**Authors:** Marie Boulain, Didier Morin, Laurent Juvin

**Affiliations:** Univ. Bordeaux, CNRS, INCIA, UMR 5287, 33000 Bordeaux, France

**Keywords:** Nanoparticles, TiO2, Mouse, Respiration, Maternal exposure

## Abstract

**Background:**

The utilization of titanium dioxide nanoparticles (TIO2NPs) has experienced a significant surge in recent decades, and these particles are now commonly found in various everyday consumer products. Due to their small size, TIO2NPs can penetrate biological barriers and elicit adverse interactions with biological tissues. Notably, exposure of pregnant females to TIO2NPs during the perinatal period has been shown to disrupt the growth of offspring. Furthermore, this exposure induces epigenetic modifications in the DNA of newborns, suggesting the possibility of multigenerational effects. Thus, perinatal exposure to TIO2NPs may induce immediate metabolic impairments in neonates, which could be transmitted to subsequent generations in the long term.

**Results:**

In this study, we utilized perinatal exposure of female mice to TIO2NPs through voluntary food intake and observed impaired metabolism in newborn male and female F1 offspring. The exposed newborn mice exhibited reduced body weight gain and a slower breathing rate compared to non-exposed animals. Additionally, a higher proportion of exposed F1 newborns experienced apneas. Similar observations were made when the exposure was limited to the postnatal period, highlighting lactation as a critical period for the adverse effects of TIO2NPs on postnatal metabolism. Importantly, the breathing deficits induced by TIO2NPs were transmitted from F1 females to the subsequent F2 generation. Moreover, re-exposure of adult F1 females to TIO2NPs exacerbated the breathing deficits in newborn F2 males.

**Conclusions:**

Our findings demonstrate that perinatal exposure to TIO2NPs disrupts postnatal body weight gain and respiration in the offspring, and these deficits are transmissible to future generations.

**Supplementary Information:**

The online version contains supplementary material available at 10.1186/s11671-023-03927-0.

## Introduction

The unique physicochemical properties of nanoparticles (NPs), attributed to their small size (below 100 nm), have led to a substantial increase in their production and utilization in various industries. Titanium dioxide nanoparticles (TIO2NPs), for instance, are extensively used in consumer products due to their whitening and photocatalytic properties. These particles can be found in everyday items such as paint, food, sunscreen, pharmaceuticals, toothpaste, cosmetics, and paper [1–3]. As a result, human exposure to TIO2NPs and their potential toxic effects can occur throughout their lifespan, spanning multiple generations. Over the past decade, numerous studies have raised concerns about the health risks associated with TIO2NPs exposure, particularly during fetal development [4]. Recent findings have demonstrated the ability of food-grade TIO2NPs to accumulate and cross the placental barrier in humans [5]. Similarly, in mice prenatal exposure to TIO2NPs has been show to damage the placental barrier by altering the proliferation, apoptosis, and vascularization of the placenta [6, 7]. Moreover, prenatal exposure to TIO2NPs has been linked to pregnancy complications [6, 8], and metabolic impairments in both the dam and the offspring [9, 10].

Apart from their prenatal toxic effects, TIO2NPs, when administered during lactation, can accumulate in the mammary gland tissue of dams, leading to pathological damages such as oxidative stress. This accumulation can disrupt the blood-milk barrier and subsequently result in inadequate body growth of the offspring [6, 11–13]. Consequently, when compared to prenatal exposure, perinatal exposure to TIO2NPs (during both gestation and lactation) could be expected to have a more complex impact on the development of the offspring acting both during the gestation and the lactation periods. In a previous study, we demonstrated that prenatal exposure to TIO2NPs altered postnatal breathing in the offspring. Newborns exhibited abnormally elevated breathing frequency, accompanied by impaired respiratory network regulation of breathing frequency [14]. However, the consequences of perinatal exposure to TIO2NPs on neonatal respiration, which more closely resembles the exposure experienced by pregnant women, remain unknown.

In this study, we conducted whole-body plethysmography recordings to monitor respiratory frequency and the occurrence of apneas in newborn mice from postnatal day 1 to 11. Compared to non-exposed animals, newborn mice perinatally exposed to TIO2NPs from the first day of gestation until weaning exhibited lower weight, slower breathing rate, and a higher likelihood of experiencing apneas. Similar results were observed when exposure was limited to the lactation period. Furthermore, following perinatal exposure of F0 dams, F1 females were bred to investigate the multigenerational inheritance of breathing alterations in the F2 generation. In the absence of a second perinatal exposure in F1 pregnant females, newborn F2 mice displayed similar breathing deficits as the F1-exposed pups. Moreover, consecutive exposures of F0 and F1 pregnant mice exacerbated the respiratory deficits in newborn F2 males. In conclusion, perinatal exposure to TIO2NPs alters postnatal respiration and body growth of the offspring over multiple generations.

## Materials and methods

### Ethical approval

All procedures were conducted in accordance with the local ethics committee of the University of Bordeaux (APAFIS#11,978–2,017,103,012,063,751) and the European Communities Council Directive (2010/63/EU). Experiments were performed on OF1 mice that were obtained from our laboratory’s breeding facility. Newborns had full access to their mother’s milk, and the mothers were fed ad libitum with full access to water. Every effort was made to minimize suffering and the number of animals used.

### Maternal exposure to TIO2NPs

To generate the F1 offspring, 2 groups of F0 pregnant mice ingested a daily dose of TIO2NPs (100 mg/kg, n = 3; 200 mg/kg, n = 3) that was mixed with chocolate spread (500 mg/kg), and administered by voluntary food intake, from the first gestational day (determined by the presence of a vaginal plug the morning following the mating night) that was maintained until weaning. 2 other groups of F0 dams received either a daily dose of chocolate spread (500 mg/kg, n = 5) alone or neither of those (n = 3). A 5th group of F0 females (n = 3) was exposed to TIO2NPs (200 mg/kg) during the lactation period (P0-21).

To generate the F2 offspring, F1 adult females that originated from perinatally-exposed litters (TIO2NPs at 200 mg/kg), were mated with non-exposed males. Then these F1 females were either exposed to TIO2NPs (200 mg/kg, n = 2, Fig. 8A), or received chocolate spread alone (500 mg/kg, n = 2, Fig. 7A), from the first gestational day, until weaning.

Pregnant mice were weighed daily, as were newborns from birth until the 11th day of life. The litter size was systematically reduced to 12 animals. TIO2NPs were purchased from Sigma-Aldrich (ref. 718,467).

### Whole body plethysmographic recordings

The breathing rate was measured using a whole-body plethysmograph (Emka Technologies) during a 5-min session [14]. Newborns were placed in a 50 ml plethysmography chamber in which the air was renewed continuously (0.8 L/min). A heating lamp was used to maintain a constant (24°C) temperature within the recording chamber. Variation of pressure within the chamber was measured at a sampling rate of 1 kHz using Iox2 software (Emka Technologies). Recordings were analyzed using Spike2 software (Cambridge Electronic Design). Apneas were considered as cessations of breathing that last during at least 3 times the mean breathing period [15].

### Physiologically based pharmacokinetic (PBPK) model

To estimate the tissue distribution of TiO_2_NPs, we used a physiologically-based pharmacokinetic model that predicts the absorption, distribution, metabolism, and excretion (ADME) of TiO_2_NPs [16]. The model considers the route of exposure (oral), animal model (mice), treatment duration (41 days), daily dose administered (100–200 mg/kg), as well as a set of parameters such as the gastrointestinal absorption rate.

### Statistical analysis

Group values were expressed as means ± SD. Differences between means were analyzed using SigmaPlot 11.0 (Systat) and assessed by Chi-square when looking at proportions. Either ANOVA on ranks followed by a Dunn’s post hoc test or ANOVA followed by a Tukey’s post hoc test was used when comparing two groups of non-parametric or parametric sets of data, respectively. Two-way and three-way MANOVA, as well as Tukey's post hoc tests, were utilized when comparing more than two groups and to assess interactions between independent variables. Differences in mean values for each parameter were taken to be significant at *p* < 0.05.

## Results

### Mice gestation is not affected by the administration of TIO2NPs

**Fig. 1:**
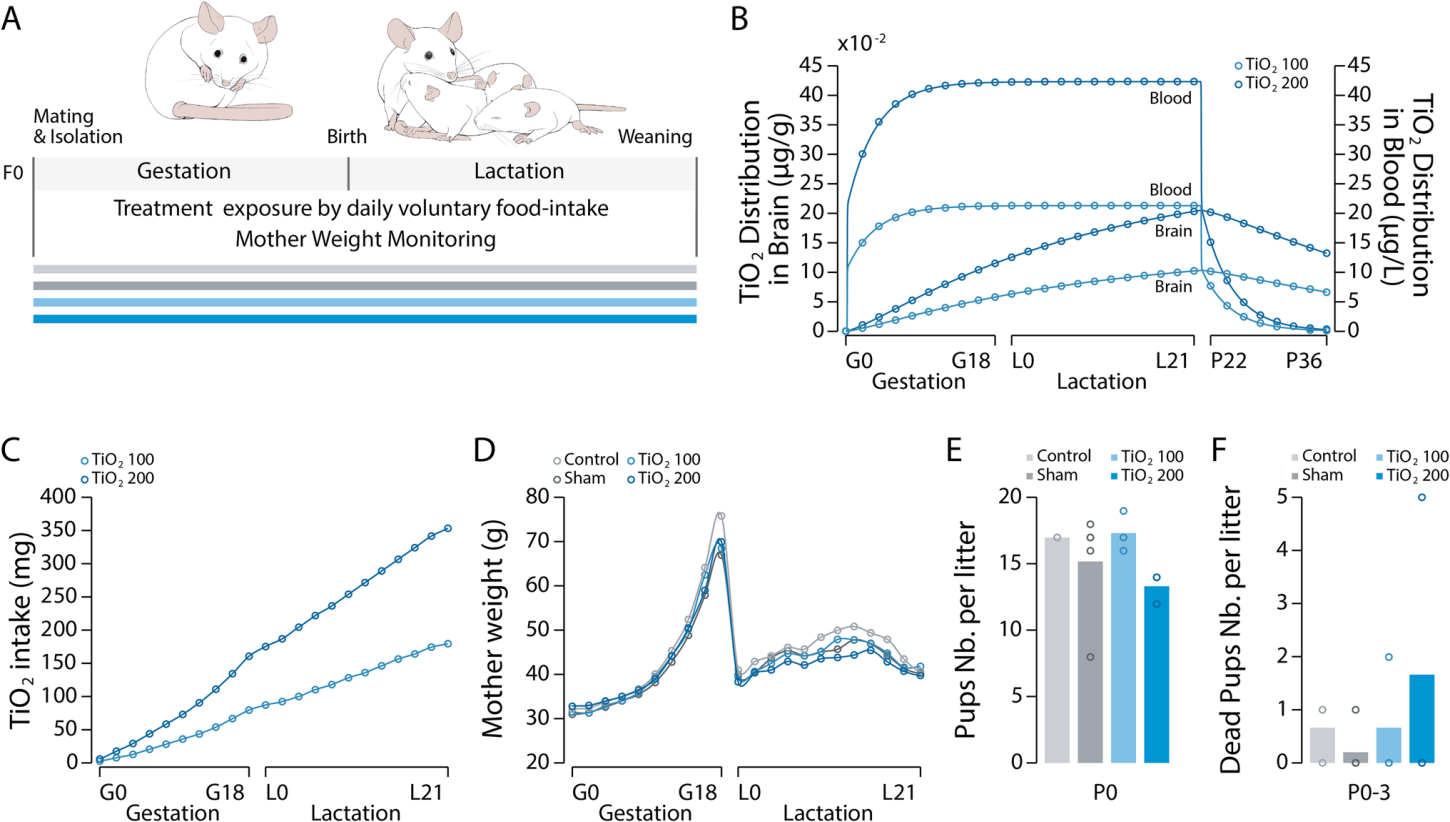
Mouse gestation is not affected by TIO2NPs administration. **A**, Schematic representation of the experimental protocol. **B**, Evolution of the predicted concentrations of TIO2NPs in the brain and blood of exposed dams during the gestation and lactation periods. **C**, Cumulative curve illustrating the quantity of TIO2NPs ingested by the dams during the gestation and lactation periods for the mice exposed to TIO2NPs at 100 mg/kg (light blue dots) and 200 mg/kg (blue dots). **D**, Scatter plot illustrating the evolution of mouse weight during the gestation and lactation. **E–F**, Bar charts illustrating the mean number of pups per litter (**E**) and the number of premature deaths (**F**) for each group of animals. For each group we used the following number of litters: control, n = 3; sham, n = 5; TiO2_100, n = 3; TiO2_200, n = 3

Two groups of pregnant mice were exposed to daily doses of P25 titanium dioxide nanoparticles (TIO2NPs) at 100 mg/kg and 200 mg/kg from the first day of gestation until the last day of lactation and weaning of the offspring (Fig. 1A). The European Food Safety Authority estimated that, on average, adults are exposed to 0.3–3.8 mg TiO2/kg body weight per day [17]. The highest daily dose used in our study was approximatively 50-fold greater than that estimated in humans, however the total amount of TIO2NPs delivered to a pregnant mouse over the ~ 19 day gestation was effectively 500-fold less than the estimated quantity taken in by a woman during the entire duration of her pregnancy (~ 280 days). The TIO2NPs used in our study were previously characterized with an average size of 25 nm, a composition of 80% anatase and 20% rutile, and a zeta potential of − 25 mV [14]. TIO2NPs were administered as a food additive in a low dose of palatable food (chocolate spread: 500 µg/g) using voluntary food intake. In comparison, a third group (sham group) received a daily dose of chocolate spread alone (500 µg/g), and a fourth group (Control group) received neither (Fig. 1A). Between the first day of gestation (G0) and the last day of lactation (P21), mice exposed to TIO2NPs at 100 mg/kg and 200 mg/kg ingested a total dose of 180.4 ± 5.9 mg and 353.7 ± 4.6 mg of TIO2NPs, respectively (Fig. 1A, C). We utilized a physiologically based pharmacokinetic (PBPK) model to evaluate the tissue distribution of TIO2NPs in the exposed mothers [16]. The model predicted that the intake of TIO2NPs at both concentrations (100 mg/kg and 200 mg/kg) would lead to a rapid increase in the blood levels of TIO2NPs, followed by a plateau (from 0.305 µg/L at G2 to 0.420 µg/L at G14 for a dose of 200 mg/kg). This rise would then be followed by a decrease after the end of the treatment due to the excretion of TIO2NPs in both urine and feces (Figure S1). The model also predicted a gradual increase in the concentration of TIO2NPs in brain tissue during the gestational and lactation periods, from 0.011 µg/g at G2 to 0.202 µg/g at L21 in females exposed to a daily dose of 200 mg/kg (Fig. 1B). A similar increase in TIO2NPs levels was also predicted by the model in various organs such as the kidneys, spleen, stomach, and intestines (Figure S1). Previous studies have reported an accumulation of TIO2 in the placenta of mice exposed to TIO2NPs [6, 8]. These accumulations coincided with changes in placental morphology, including modifications in weight, size, vascularization, and an ionic imbalance in the maternal serum. These alterations resulted in pregnancy complications, ranging from reduced fetal body weight to a reduced number of viable fetuses [6–8, 18].

**Fig. 2:**
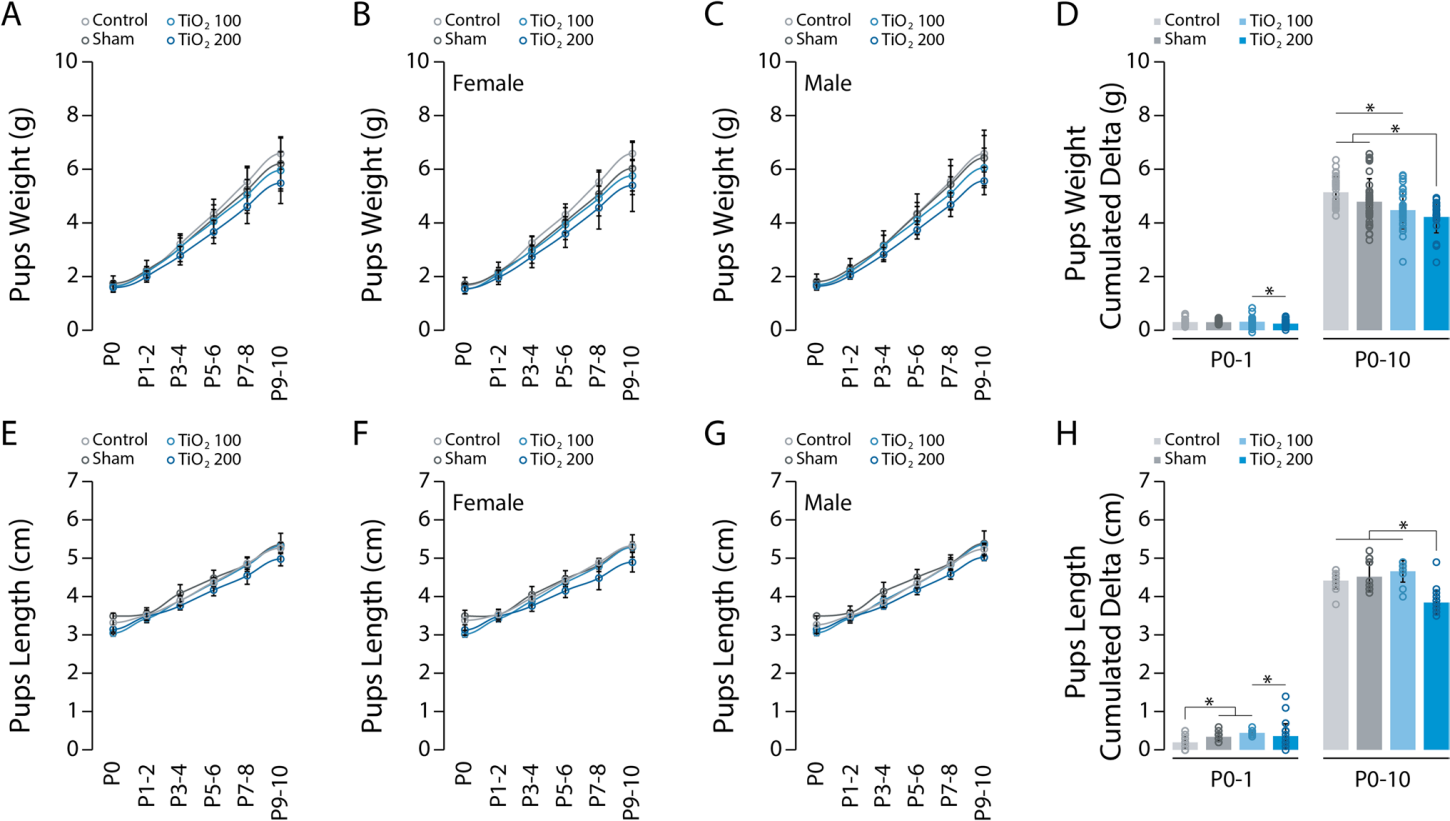
Evolution of Pups’ Weight and Body Length. **A**, Scatter plot illustrating the evolution of body weight over the P0-10 period for control (light grey), sham (dark grey), TiO2_100 (light blue), and TiO2_200 (blue) pups. **B, C**, Same representation as in A for female (**B**) and male pups (**C**). **D**, Bar chart illustrating pups’ weight gain during the P0-1 and P0-10 periods. **E–G**, Same representation as in A-C for pups’ growth curve. **H**, Bar chart illustrating pups’ length gain during the P0-1 and P0-10 periods. * p < 0.05. For each group we used the following number of pups: control, n = 35, females = 16, males = 19; sham, n = 55, females = 31, males = 24; TiO2_100, n = 34, females = 11, males = 23; TiO2_200, n = 36, females = 19, males = 17

To assess the possibility of abnormal pregnancy outcomes, we conducted daily measurements of the gestational weight gain of the pregnant mice (Fig. 1D). Both exposed and non-exposed females showed similar weight gains during pregnancy and the postnatal period, indicating no apparent effect of TIO2NPs exposure. Additionally, all four groups of animals had similar litter sizes (Fig. 1E) and a similar mortality rate among newborns (Fig. 1F). We also monitored the weight gain and body length of the pups from the first day of life (P0) until the 11th day (P10). At birth, animals from all four groups had similar body weights (ANOVA on ranks, H = 4.182, *p* = 0.243; Fig. 2A). However, differences were observed between the four groups in the following days. For instance, at P9-10, significant differences were found between the control and sham groups, the TiO2_100 and TiO2_200 groups, and the sham and TiO2_200 groups (ANOVA on ranks, H = 31.380, *p* < 0.001; Fig. 2A). This was evident when analyzing the cumulative weight gain of the pups between P0 and P10. The exposed pups exhibited significantly lower weight gain compared to the control and sham pups (P0-10: ANOVA on ranks, H = 27.182, *p* < 0.001; Fig. 2D). We further investigated whether TIO2NPs affected the weight of the pups differently based on their sex. Both exposed females (Fig. 2B) and males (Fig. 2C) showed reduced weight gain compared to the non-exposed pups (Females: P9-10: ANOVA on ranks, H = 17.969, *p* < 0.001; Males: P9-10: ANOVA on ranks, H = 17.081, *p* < 0.001). At birth, both TiO2_100 and TiO2_200 animals exhibited shorter body length compared to the control and sham groups (ANOVA on ranks, H = 42.953, *p* < 0.001; Fig. 2E). However, at P9-10, only the TiO2_200 animals were significantly shorter than the control and sham groups (ANOVA on ranks, H = 44.200, *p* < 0.001; Fig. 2E-G). Overall, the TiO2_200 animals showed reduced body growth compared to the other groups (P0-10: ANOVA on ranks, H = 34.460, *p* < 0.001; Fig. 2H). In conclusion, exposure to TIO2NPs, particularly at a dose of 200 mg/kg, alters the body growth of newborn animals during the P0-10 period. Indeed, despite numerous studies on the subject, it remained unclear whether TIO2NPs perinatal exposure affects the growth of neonates. Previous studies have focused the weight gain of newborns following prenatal exposure to TiO_2_, some reporting no significant changes [10, 19–21], while others indicated a notable reduction in pups’ weight [6, 22, 23]. These discrepancies can be attributed to various factors, such as the use of different animal models (mice vs. rats), variations in the size of TIO2NPs (ranging from 6.5 to 25 nm), differences in the route and dosage of administration (gavage, inhalation, i.v.), and variations in the frequency of administration (single, repeated, or daily). Similarly, comparable observations were made regarding the administration of TIO2NPs during lactation, with some studies demonstrating reduced offspring body weight [12, 13], while others showed no effect [11]. In this context, our findings provide new insights into the effects of perinatal exposure to TIO2NPs on neonatal growth. In a previous study, we used the same TIO2NPs, with the same number, route, and dosage of administration, but limited the exposure to the gestation period. In a previous study, we observed no changes in the body weight of the pups that were exposed to TIO2NPs through their mother's diet during gestation [14]. Conversely, in the present study, we demonstrate a reduced body weight gain in pups exposed to TIO2NPs during lactation (Fig. 2, S2 and Table 1). Consequently, our studies indicate that postnatal, but not prenatal, oral exposure to P25 TIO2NPs alters neonatal body growth, likely through the disruption of breastfeeding. Previous research has shown that the administration of TIO2NPs during the lactation period leads to the accumulation of these particles in the mammary gland, causing pathological damage to the tissue and disruption of the blood-milk barrier [11–13]. Notably, the mammary gland exhibited increased oxidative stress, apoptosis rate, and loss of tight junctions. While the milk yield and nutrient quality were not directly affected, the concentration of TIO2NPs and ROS in the dams' milk increased.

**Table 1:** Litter size, female-to-male pup ratio, pup length, and pup weight at P0

	CTRL	SHAM	TiO2_100	TiO2_200	TiO2_200_Lact	F2 Sham	F2 TiO2_200
Litter size	17.00	15.20	17.33	13.33	15.33	16.50	13.00
SD	0.00	4.09	1.53	1.15	2.31	2.12	2.83
Female/male ratio	0.84	1.24	0.57	1.12	1.42	1.18	1.30
Pup length (cm)	3.33	3.50	3.05	3.14	3.22	3.21	3.29
SD	0.25	0.16	0.08	0.11	0.08	0.14	0.14
Pup weigth (g)	1.68	1.76	1.65	1.61	1.73	1.78	1.76
SD	0.10	0.27	0.20	0.18	0.12	0.10	0.11

### Perinatal exposure to TIO2NPs impairs offspring breathing rhythmicity

**Fig. 3:**
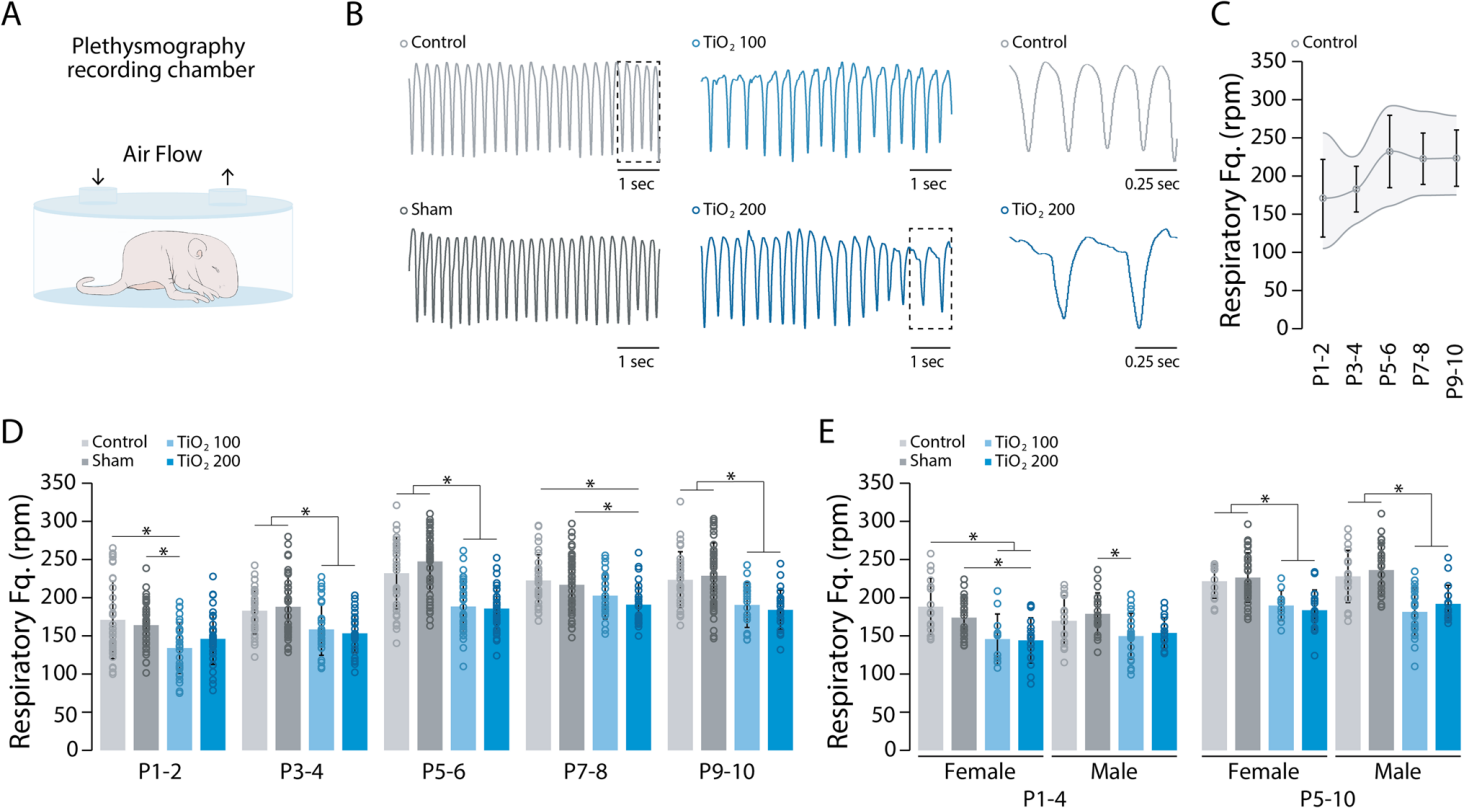
Perinatal Exposure to TIO2NPs Impairs Breathing Rhythmicity of Offspring. **A**, Schematic representation of the plethysmography recording chamber. **B, Left**, Plethysmographic recordings at P6 of pups breathing (control, light grey; sham, dark grey; TiO2_100, light blue; and TiO2_200, blue). **B, Right**, Expanded portion (indicated by dotted lines) of control and TiO2_200 traces. **C**, Scatter plot illustrating the evolution of the breathing mean frequency of control animals during the P1-10 period. **D**, Bar chart illustrating the evolution of breathing rate for control (light grey bars), sham (dark grey bars), TiO2_100 (light blue bars), and TiO2_200 (blue bars) pups over the P1-10 period. **E**, Bar chart illustrating the breathing rate of females and males for each group of animals during the P1-4 and P5-10 periods. *p < 0.05. For each group we used the following number of pups: control, n = 35, females = 16, males = 19; sham, n = 55, females = 31, males = 24; TiO2_100, n = 34, females = 11, males = 23; TiO2_200, n = 36, females = 19, males = 17

In the control group, the respiratory frequency of animals increased from 171.07 ± 50.82 at P1-2 to 232.13 ± 47.37 at P5-6 (MANOVA: F = 73.501, *p* < 0.001, Tukey’s test: P1-2 vs. P5-6, *p* < 0.001), after which it reached a plateau during the following days (MANOVA: F = 73.501, *p* < 0.001, Tukey’s test: P5-6 vs. P9-10, *p* > 0.05; Fig. 3C). This typical pattern of respiratory development was observed in all groups of animals (MANOVA: F = 73.501, *p* < 0.001, Fig. 3D). However, neonates from TIO2NPs-exposed litters exhibited a slower breathing rate compared to non-exposed animals (MANOVA: F = 73.501, Tukey’s test: control vs sham, *p* > 0.05; control vs TiO2_100, *p* < 0.001; control vs TiO2_200, *p* < 0.001; sham vs TiO2_100, *p* < 0.001; sham vs TiO2_200, *p* < 0.001; TiO2_100 vs TiO2_200, *p* > 0.05; Fig. 3B, D). We also observed an interaction between the treatment and the age of the animals (MANOVA: F = 2.034, *p* = 0.019), indicating a progressive and cumulative-like effect of TIONPs exposure during lactation. When examining sex differences, we observed no differences between males and females (MANOVA: F = 0.336, *p* = 0.563). Exposed newborn females had a slower breathing frequency compared to sham and control females at both P1-4 and P5-10 (ANOVA: females: P1-4: F = 7.699, *p* < 0.001; P5-10: F = 11.979, *p* < 0.001), and a similar difference was also observed between exposed and non-exposed males (ANOVA: males: P1-4: F = 4.712, p = 0.005; P5-10: F = 15.510, *p* < 0.001; Fig. 3E). Thus, our findings demonstrate that perinatal exposure to TIO2NPs leads to a slower breathing frequency in neonates compared to non-exposed animals, regardless of sex. As discussed in the above section, prenatal exposure to TIO2NPs elicits morphological alterations in the placental barrier and consequently impacts its efficacy, potentially facilitating the transfer of TIO2NPs from the mother to the fetus [6, 8, 18, 23]. Other studies notably highlighted an impairment in lung development in the offspring, characterized by inflammatory reactions at the pulmonary level [23, 24]. In a previous work we demonstrated that prenatal exposure to TIO2NPs similar to the one utilized in this study did not result in any structural modifications of the lungs: the lung/body weight ratio, lung size, and number of lung alveoli were similar between exposed and non-exposed pups [14]. Thus, it is likely that our protocol of exposure to TIO2NPs does not significantly alter the lung development of the offspring, although further complementary studies could be conducted to confirm this.

### Litters exposed perinatally to TIO2NPs have a higher frequency of apneas

**Fig. 4:**
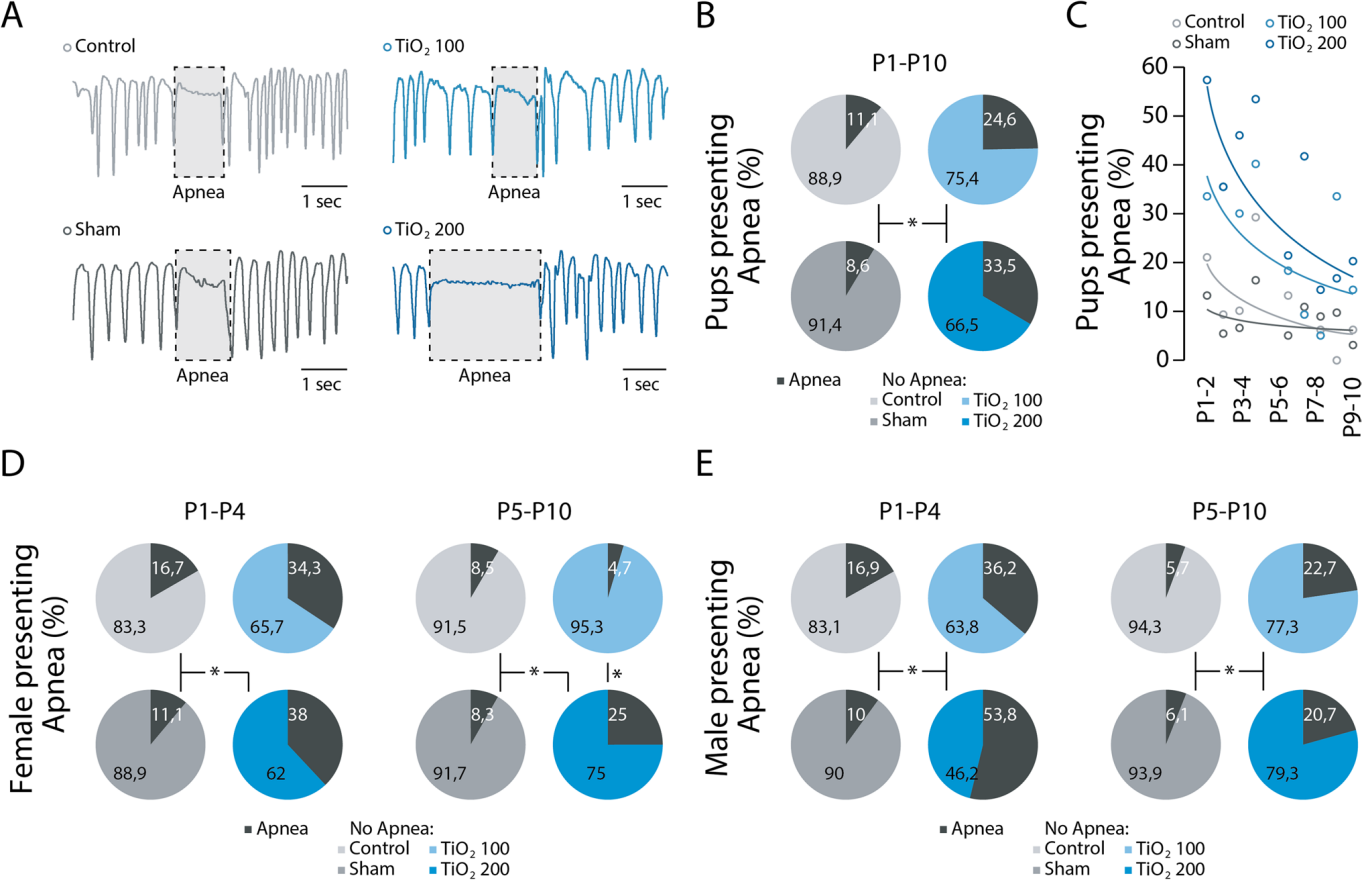
Perinatal exposure to TIO2NPs increases the incidence of apneas. **A**, Plethysmographic recordings at P6 showing the presence of apneas (indicated by the shaded box) in all 4 groups (control, light grey; sham, dark grey; TiO2_100, light blue; and TiO2_200, blue). **B**, Proportion of neonates exhibiting apneas for each group over the P1-10 period. Apneas are represented in black on pie charts. **C**, Evolution of the proportion of neonates exhibiting apneas during the P1-10 period. **D-E**, Pie charts illustrating the proportion of females (**D**) and males (**E**) exhibiting apneas for each group of animals during the P1-4 and P5-10 periods. For each group we used the following number of pups: control, n = 35, females = 16, males = 19; sham, n = 55, females = 31, males = 24; TiO2_100, n = 34, females = 11, males = 23; TiO2_200, n = 36, females = 19, males = 17

**Fig. 5:**
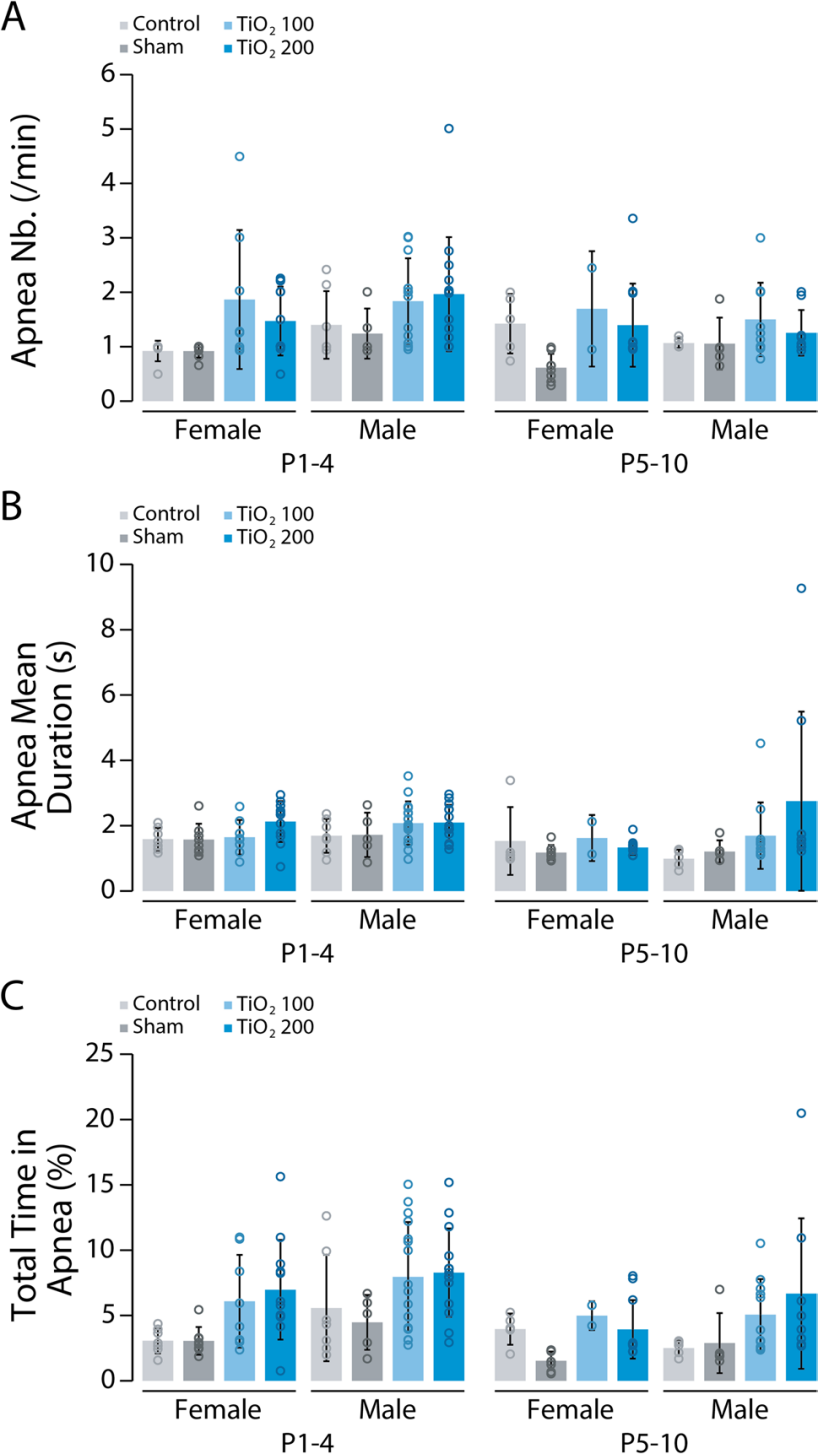
Temporal structure of apneas is not affected by TIO2NPs exposure. **A**, Bar chart illustrating the number of apneas produced by female and male animals in control (light grey), sham (dark grey), TiO2_100 (light blue), and TiO2_200 (blue) groups during the P1-4 and P5-10 periods. **B**, **C**, Same representation as in A, for the mean duration (**B**) and total time spent in apnea (**C**). *p < 0.05. For each group we used the following number of pups: control, n = 35, females = 16, males = 19; sham, n = 55, females = 31, males = 24; TiO2_100, n = 34, females = 11, males = 23; TiO2_200, n = 36, females = 19, males = 17

When examining the regularity of breathing in newborn mice, we observed that all groups of animals experienced apneas (Fig. 4A). However, the proportion of animals producing apneas was significantly higher in the TiO2-exposed groups (24.6% for TiO2_100 and 33.5% for TiO2_200) compared to the control and sham groups (11.1% and 8.6%, respectively; Chi-Squared test = 74.923, *p* < 0.001; Fig. 4B). Between P0 and P10, there was a similar trend of decreasing apnea occurrence in all groups (Fig. 4C). This trend was consistent in both female and male newborns and not specific to a particular sex (females: P1-4: Chi-Squared test = 16.675, *p* < 0.001; P5-10: Chi-Squared test = 15.482, *p* = 0.001; males: P1-4: Chi-Squared test = 34.826, *p* < 0.001; P5-10: Chi-Squared test = 17.976, *p* < 0.001; Fig. 4D). Next, we compared the temporal organization of apneas produced by animals in all four groups during the early (P1-4) and late (P5-10) postnatal periods. Males and females produced a similar number of apneas regardless of their group (Tukey’s test: males: P1-4: *p* > 0.05, P5-10: *p* > 0.05; females: P1-4: *p* > 0.05, P5-10: *p* > 0.05; Fig. 5A). We then measured the mean duration of apneas and found no differences between the four groups during both the P1-4 and P5-10 periods in females and males (Tukey’s test: males: P1-4: p > 0.05, P5-10: *p* > 0.05; females: P1-4: *p* > 0.05, P5-10: *p* > 0.05; Fig. 5B). Finally, we assessed the total time spent in apnea during the P1-4 and P5-10 periods. In both females and males, exposure to TIO2NPs did not significantly affect this parameter (Tukey’s test: males: P1-4: *p* > 0.05, P5-10: *p* > 0.05; females: P1-4: *p* > 0.05, P5-10: *p* > 0.05; Fig. 5C). Overall, our data indicate no major differences in the temporal organization of apneas among the four groups throughout the P1-10 period.

### Exposure to TIO2NPs during the lactation period impairs offspring breathing rhythmicity

**Fig. 6:**
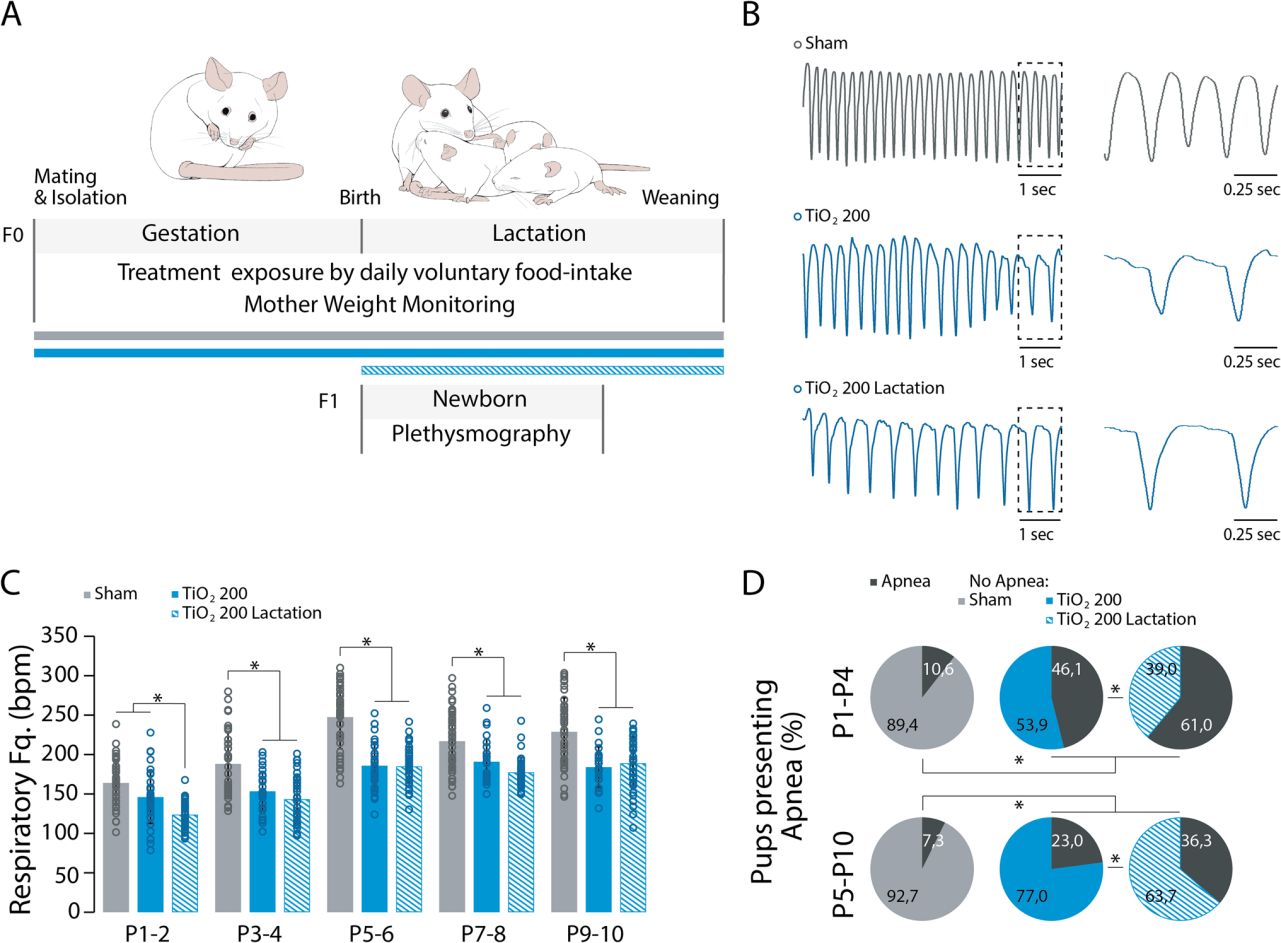
Exposure to TIO2NPs during lactation and perinatal period have similar effects on offspring breathing. **A**, Schematic representation of the experimental protocol. **B, Left**, Plethysmographic recording at P6 of sham, TiO2_200, and TiO2_200_Lact pups. **B, Right**, Expanded portion (indicated by dotted lines) of the trace on the left. **C**, Bar chart illustrating the evolution of the breathing rate of sham (dark grey bars), TiO2_200 (blue bars), and TiO2_200_Lact (blue hatched bars) pups over the P1-10 period. **D**, Pie chart representation of the number of pups exhibiting apneas during the P1-4 and P5-10 periods. Apneas are represented in black on pie charts. *p < 0.05. For each group we used the following number of pups: sham, n = 55, females = 31, males = 24; TiO2_200, n = 36, females = 19, males = 17; TiO2_200_Lact, n = 36, females = 17, males = 19

We found that the respiratory activity of newborns is affected by exposure to TIO2NPs during the perinatal period, encompassing both the pre- and postnatal stages. To investigate whether exposure limited to the postnatal period produced similar effects on respiratory function, female mice were exposed to TIO2NPs (200 mg/kg) during lactation (TiO2_200_Lact; Fig. 6A). Offspring from exposed litters exhibited reduced weight gain and shorter body length compared to non-exposed animals (Figure S2). Subsequently, we recorded the breathing patterns of TiO2_200_Lact newborn mice (Fig. 6B). At P1-2, TiO2_200_Lact pups displayed a slower breathing rate compared to TiO2_200 pups (MANOVA: F = 89.296, *p* < 0.001, Tukey’s test: P1-2: *p* < 0.05; Fig. 6C). Throughout the P1-10 period, the breathing frequency of TiO2_200_Lact pups increased until P5-6 and then stabilized at 185.3 ± 24.9 RPM, with breathing frequencies that were similar to TiO2_200 pups (MANOVA: F = 89.296, *p* < 0.001, Tukey’s test: P3-4: *p* > 0.05; P5-6: *p* > 0.05; P7-8: *p* > 0.05; P9-10: *p* > 0.05; Fig. 6C). Moreover, over the P1-10 period, TiO2_200_Lact pups exhibited a slower breathing frequency than non-exposed animals (MANOVA: F = 89.296, *p* < 0.001, Tukey’s test: P1-2: *p* < 0.05; P3-4: *p* < 0.05; P5-6: *p* < 0.05; P7-8: *p* < 0.05; P9-10: *p* < 0.05; Fig. 6C). This effect was not specific to a particular sex, as both male and female TiO2_200_Lact pups exhibited reduced breathing frequency (MANOVA: F = 50.183, *p* < 0.001; Figure S3A), but no interaction was observed between the treatment and the sex (F = 0.281, *p* = 0.755; Figure S3A).

Additionally, we observed an increased proportion of TiO2_200_Lact pups experiencing apneas compared to sham and TiO2_200 pups during both the P1-4 and P5-10 periods (P1-4: Chi-Squared test = 78.456, *p* < 0.001; P5-10: Chi-Squared test = 52.719, *p* < 0.001; Fig. 6D). However, the temporal characteristics of apneas in TiO2_200_Lact pups resembled those of TiO2_200 pups, with no significant differences in the number per minute (Tukey’s test: Female: P1-4: *p* > 0.05, P5-6: *p* > 0.05; Male: P1-4: *p* > 0.05, P5-6: *p* > 0.05; Figure S3B), mean duration (Tukey’s test: Female: P1-4: *p* > 0.05, P5-6: *p* > 0.05; Male: P1-4: *p* > 0.05, P5-6: *p* > 0.05; Figure S3C), and total time in apnea (Tukey’s test: Female: P1-4: *p* > 0.05, P5-6: *p* > 0.05; Male: P1-4: *p* > 0.05, P5-6: *p* > 0.05; Figure S3D). In summary, our findings indicate that exposure to TIO2NPs during either the perinatal or postnatal period alters the breathing of newborn mice. In a prior study, we demonstrated that mice offspring exposed to TIO2NPs during gestation exhibited an abnormally elevated breathing rate [14]. Our current findings reveal that offspring exposed to TIO2NPs during lactation experience a slower breathing rate (Fig. 3). Notably, pups exposed during lactation also exhibit reduced weight gain in the first two postnatal weeks (Fig. 2), while those exposed during gestation show normal weight gain during the same period [14]. While we cannot definitively pinpoint the source of these differences at this time, it is crucial to recognize that prenatal exposure primarily impacts respiration, whereas lactational exposure appears to have a more wide-ranging influence on pup physiology. The timing of exposure is known to play a significant role in potential outcomes, encompassing a range of physiological abnormalities. Indeed, the consequences of TIO2NPs exposure seem to vary with age, as younger rats display increased sensitivity to TIO2NPs toxicity compared to adult animals [27]. For example, in humans, distinct cognitive deficits are associated with prenatal and postnatal exposure to cannabis [25]. The heightened impact of cannabis during early childhood exposure may be attributed to the underdeveloped detoxification pathways at this stage of development [26]. Taken together our findings suggest that that the timing of exposure to TIO2NPs has distinct effects on respiratory function and overall physiology in mouse offspring, emphasizing the importance of considering exposure windows when assessing potential developmental outcomes.

In comparison to humans, mice have an immature respiratory system at birth, and the presence of apneas has been associated with immature breathing, as observed in preterm infants [28–30]. Thus, our results suggest a delayed maturation of the respiratory function in exposed pups that could be related to a metabolic distress [6]. Notably, the maternal and pup's diet directly influences offspring body weight and breathing [31–33]. Interestingly, newborn mice subjected to fasting (3–6 h) exhibited breathing instability characterized by decreased breathing frequency and increased occurrence of apneas [33]. Despite these important findings, the mechanisms linking impaired neonatal growth and breathing alterations remain unclear. TIO2NPs can directly affect developmental processes such as cell proliferation, apoptosis, neurogenesis, and neuron-glial interactions, all of which are crucial for neural circuit formation [22, 34–36]. In a previous study, we demonstrated that prenatal exposure to TIO2NPs impairs the development and function of the primary inspiratory center, specifically the pre-Bötzinger complex [14, 37]. Postnatal exposure to TIO2NPs may also disrupt the functioning of other brainstem nuclei, including the retrotrapezoid nucleus and the parafacial respiratory group (RTN/pFRG). Mutations in the Phox2b gene, for instance, result in reduced neuronal density in the RTN/pFRG complex and a hypoventilation syndrome in offspring [38]. Additionally, it is plausible that TIO2NPs exposure indirectly impairs breathing by dysregulating neurotransmitter systems. Studies in the central nervous system of rats have shown that acute administration of TIO2NPs decreases serotonin levels [39, 40], a key neuromodulator in the neural centers that regulate breathing frequency [41, 42]. Notably, neonates lacking serotoninergic neurons display a higher likelihood of experiencing apneas and severe hypoventilation syndrome [43]. Importantly, newborns with serotonin neuron deficiencies also exhibit associated deficits in body growth until the end of the second week of life.

### Multigenerational consequences of TIO2NPs exposure on offspring breathing

**Fig. 7:**
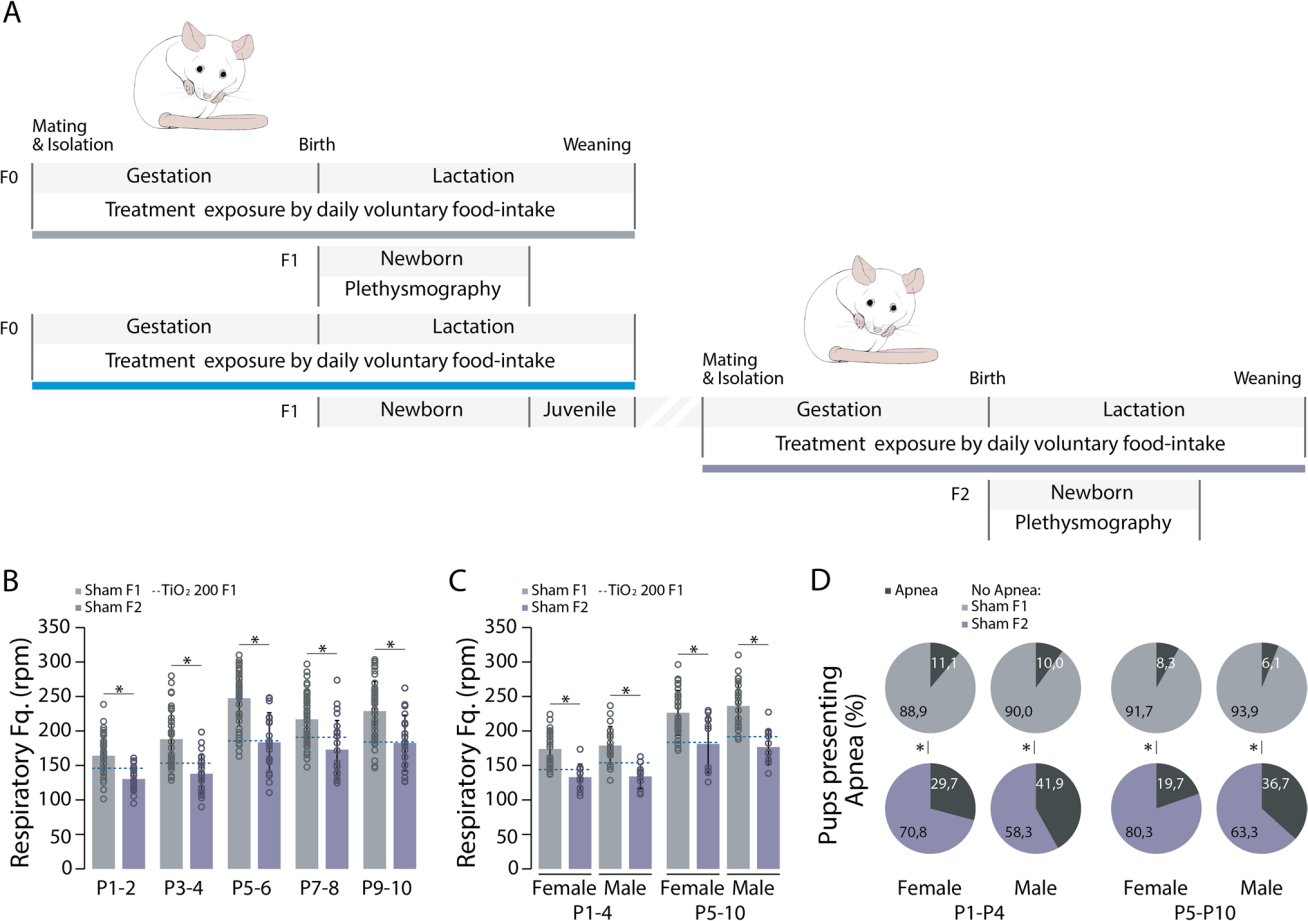
Transmission of TIO2NPs-induced breathing alterations to F2 sham.** A**, Schematic representation of the experimental protocol. **B**, Bar chart illustrating the evolution of the breathing rate of F1 sham (dark grey bars) and F2 sham (purple bars) pups over the P1-10 period. The blue dotted lines indicate the mean values of F1 TiO2_200 animals. **C**, Bar chart illustrating the breathing rate of females and males for each group of animals during the P1-4 and P5-10 periods. The blue dotted lines indicate the mean values of F1 TiO2_200 animals. **D**, Proportion of female and male neonates producing apneas for each group over the P1-4 and P5-10 periods. Apneas are indicated in black on pie charts. *p < 0.05. For each group we used the following number of pups: F1 sham, n = 55, females = 31, males = 24; F2 sham, n = 24, females = 12, males = 12

We aimed to investigate whether the breathing alterations observed in exposed animals during the neonatal period could be transmitted to subsequent generations. To explore this, we bred adult females (F1) from F1 TiO2_200 litters with non-exposed males and monitored the respiratory activity of their offspring (F2 sham) during the P1-10 period (Fig. 7A). F2 sham pups exhibited a slower breathing rate compared to F1 sham pups (MANOVA: F = 85.489, *p* < 0.001, Tukey’s test: P1-2: *p* < 0.05; P3-4: *p* < 0.05; P5-6: *p* < 0.05; P7-8: *p* < 0.05; P9-10: *p* < 0.05; Fig. 7B). This effect was observed both in females and males (Tukey's test: Females: P1-4: *p* < 0.05, P5-10: *p* < 0.05; Males: P1-4: *p* < 0.05, P5-10: *p* < 0.05; Fig. 7C), with no interaction between sex and treatment (MANOVA: F = 0.743, *p* = 0.390; Fig. 7C). Additionally, compared to F1 sham pups, there was a significantly higher proportion of F2 sham female and male pups experiencing apneas (Females: P1-4: Chi-Squared test = 5.531, *p* = 0.019; P5-10: Chi-Squared test = 3.951, *p* = 0.047; Males: P1-4: Chi-Squared test = 13.899, *p* < 0.001; P5-10: Chi-Squared test = 22.056, *p* < 0.001; Fig. 7D). Importantly, the temporal characteristics of apneas were not significantly different between the two groups (Figure S4). These alterations in breathing frequency were accompanied by a reduced growth rate in the exposed pups (Figure S5 and Table 1).

**Fig. 8:**
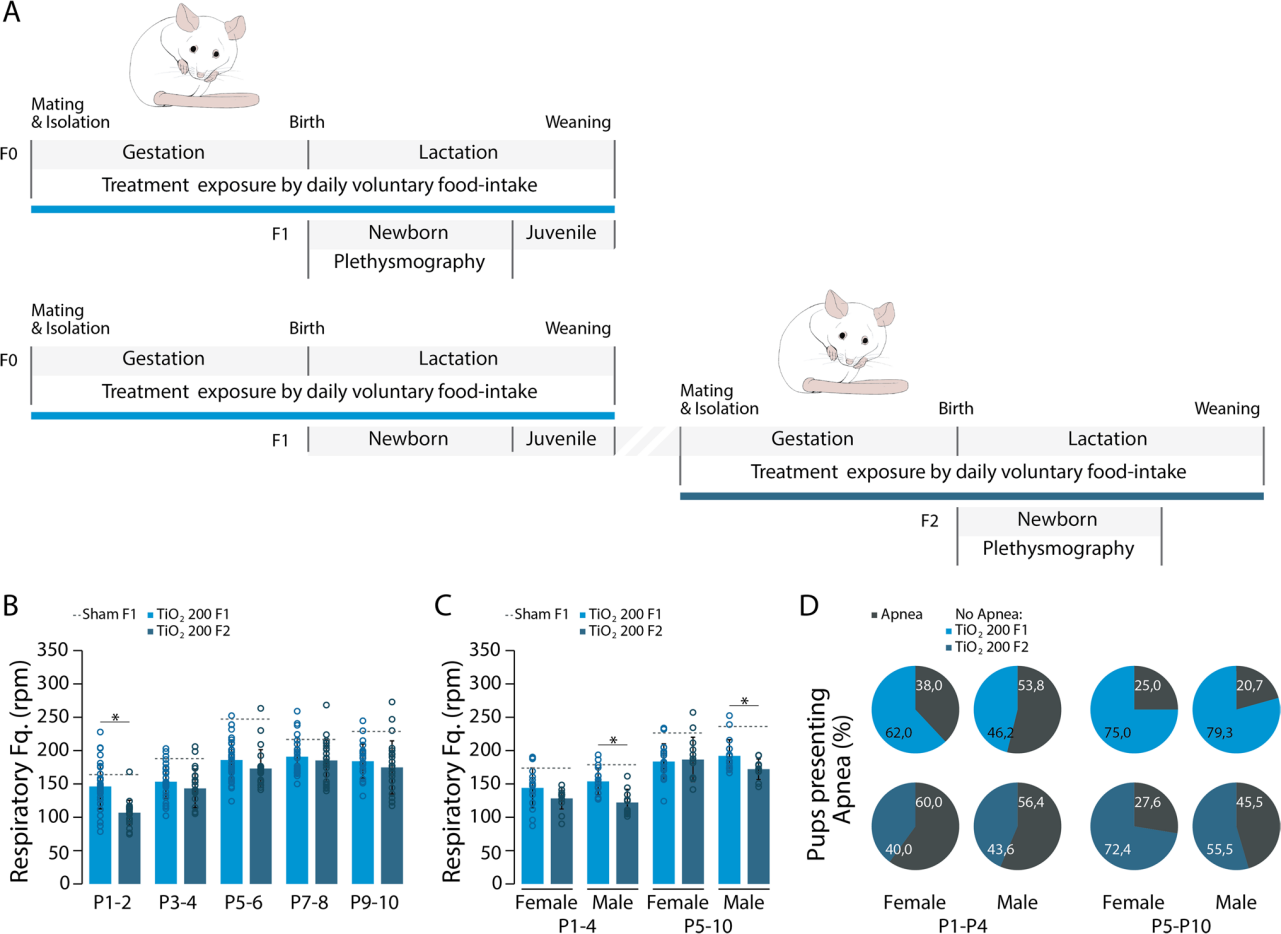
Transmission and exacerbation of TIO2NPs-induced breathing alterations by TiO2 re-exposure in F2 females and males, respectively. **A**, Schematic representation of the experimental protocol. **B**, Bar chart illustrating the evolution of the breathing rate of F1 TiO2_200 (blue bars) and F2 TiO2_200 (dark blue bars) pups over the P1-10 period. The grey dotted lines indicate the mean values of F1 sham animals. **C**, Bar chart illustrating the breathing rate of females and males for each group of animals during the P1-4 and P5-10 periods. The grey dotted lines indicate the mean values of F1 sham animals. **D**, Proportion of female and male neonates producing apneas for each group over the P1-4 and P5-10 periods. Apneas are indicated in black on pie charts. *p < 0.05. For each group we used the following number of pups: F1 TiO2_200, n = 36, females = 19, males = 17; F2 TiO2_200, n = 24, females = 13, males = 11

To examine whether the multigenerational effects of TIO2NPs exposure could render F2 pups more vulnerable to re-exposure, adult females (F1) derived from TiO2_200 litters were bred with non-exposed males and subsequently re-exposed to a daily dose of TIO2NPs at 200 mg/kg from the first day of gestation until weaning (Fig. 8A). Re-exposed pups (F2 TiO2_200) displayed a diminished growth rate compared to F1 TiO2_200 pups (Figure S6 and Table 1). Throughout the P1-10 period, with at the exception of P1-P2, the breathing frequency of F2 TiO2_200 pups was similar to that of F1 TiO2_200 pups (MANOVA: F = 11.246, *p* < 0.001, Tukey’s test: P1-2: *p* < 0.05; P3-4: *p* > 0.05; P5-6: *p* > 0.05; P7-8: *p* > 0.05; P9-10: *p* > 0.05; Fig. 8B). Importantly, when examining possible sex differences, we observed an interaction between sex and treatment (MANOVA: F = 4.286, *p* = 0.041; Fig. 8C), re-exposure to TIO2NPs resulted in a reduction of the breathing rate specifically in F2 male pups, but not in F2 female pups, compared to F1 TiO2_200 pups during both the P1-4 and P5-10 periods (Tukey's test: Females: P1-4: *p* > 0.05, P5-10: *p* > 0.05; Males: P1-4: *p* < 0.05; P5-10: *p* < 0.05; Fig. 8C). Additionally, similarly to TiO2_200 pups, we observed a high proportion of F2 TiO2_200 males and females experiencing apneas during the P1-4 period (F2 TiO2_200 Females: 60%; F2 TiO2_200 Males: 56.4; F2 vs F1 Females: Chi-Squared test = 4.002, *p* = 0.045; F2 vs F1 Males: Chi-Squared test < 0.001, *p* = 0.976; Fig. 8D), and this effect persisted for males but not females during the P5-10 period (F2 TiO2_200 Females: 27.6%; F2 TiO2_200 Males: 45.5%; Females: Chi-Squared test = 0.014, *p* = 0.906; Males: Chi-Squared test = 6.023, *p* = 0.014; Fig. 8D). Furthermore, the temporal structure of apneas was similar between F1 TiO2_200 and F2 TiO2_200 pups (Figure S7).

These results demonstrate the long-lasting multigenerational adverse effects of TIO2NPs exposure, highlighting a potential vulnerability of F2 males to re-exposure. Previous studies have indicated that inhalation exposure of pregnant rat to TIO2NPs can lead to persistent alterations in oxidative species production across multiple generations, from F0 dams to F2 offspring [44]. These alterations were associated with reduced weight in the F1 and F2 generations and changes in metabolism, including increased glycemia. The perinatal exposure to TIO2NPs induces an early childhood stress response due to its various consequences on F1 newborns, such as reduced weight and breathing rate (Figs. 2–4). Notably, metabolic disturbances during the perinatal period have been shown to induce epigenetic modifications with potential implications for subsequent generations [33, 45]. The increase in oxidative species production triggered by TIO2NPs exposure can disrupt gene expression and DNA methylation [46]. The small size and high reactivity of TIO2NPs enable them to induce DNA methylation in cultured cell lines [47–53], and as they can cross biological barriers, they can be transferred from the mother to the offspring, where they can impact DNA methylation. Indeed, maternal exposure to TIO2NPs can directly cause epigenetic modifications in the DNA of the progeny [21, 54–56]. These studies have reported genome-wide alterations in DNA methylation in the brains and cardiac tissues of offspring, leading to impairments in apoptosis, cell cycle regulation, mitochondrial function, DNA repair, and inflammation-related pathways.

Furthermore, we observed a notable susceptibility of F2 male pups to perinatal re-exposure to TIO2NPs compared to females. Previous research has reported sex-dependent epigenetic alterations following prenatal TIO2NPs exposure. Specifically, DNA methylation was found to be increased in 614 genes and decreased in 2924 genes in male offspring, while in female offspring, DNA methylation was increased in 6220 genes and decreased in 6477 genes [21]. These findings provide a potential explanation for the sex-specific impact of TIO2NPs re-exposure, particularly affecting F2 males (Fig. 8C, D). Although a direct connection between specific epigenetic modifications and respiratory function cannot be established at present, a recent study demonstrated that re-exposure to nicotine in F1 animals, previously exposed during F0 gestation, enhanced the asthmatic phenotype in the F2 generation, primarily in males [57]. Once again, these sex-associated phenotypes were linked to sex-specific DNA methylation patterns.

## Conclusion

In conclusion, our findings demonstrate that perinatal exposure to TIO2NPs disrupts neonatal respiration throughout multiple generations, thereby establishing a susceptibility to TIO2NPs re-exposure in subsequent pregnancies. We hypothesize that sex-specific epigenetic modifications may underlie the persistent deficits observed across generations, although the precise targets of TIO2NPs remain to be identified. These results raise concerns regarding the transmission of neurophysiological impairments associated with adverse maternal environments, particularly in the context of nanoparticles.

### Supplementary Information

Figure S1: Evolution of tissue concentration of TIO2NPs in exposed dams over gestation and lactation periods. Evolution of TIO2NPs concentration in exposed dams in faeces (A), urine (B), stomach and intestines (C), kidneys, liver, and spleen (D), lung (E), and heart (F), for mice exposed to TIO2NPs at 100 mg/kg (light blue dots) and 200 mg/kg (blue dots) during gestation and lactation periods (PNG 542 KB)

Figure S2: Evolution of pups’ weight and body length following TIO2NPs exposure during lactation. A, Scatter plot illustrating the evolution of body weight of sham (dark grey), TiO2_200 (blue), and TiO2_200 Lact (hatched blue) pups over the P0-10 period. B, C, Same representation as in A, for females (B) and males (C). D, Bar chart illustrating pups’ weight gain during the P0-1 and P0-10 periods. E-G, Same representation as in A-C of pups’ growth curves. H, Bar chart illustrating pups’ length gain during the P0-1 and P0-9 periods. * p < 0.05. For each group we used the following number of pups: sham, n = 55, females = 31, males = 24 ; TiO2_200, n = 36, females = 19, males = 17 ; TiO2_200_Lact, n = 36, females = 17, males = 19 (PNG 343 KB)

Figure S3: Apneas temporal structure is not affected by TIO2NPs exposure during lactation. A-D, Bar charts illustrating the breathing rate of females and males (A), the number of apneas (B), the mean duration of apneas (C), and the total time spent in apnea (D) of sham (dark grey bars), TiO2_200 (blue bars), and TiO2_200_Lact (blue hatched bars) pups during the P1-4 and P5-10 periods. * p < 0.05. For each group we used the following number of pups: sham, n = 55, females = 31, males = 24 ; TiO2_200, n = 36, females = 19, males = 17 ; TiO2_200_Lact, n = 36, females = 17, males = 19 (PNG 314 KB)

Figure S4: Apneas temporal structure is altered in F2 sham pups. A-C, Bar charts illustrating the number of apneas (A), the mean duration of apneas (B), and the total time spent in apnea (C) of F1 sham (dark grey bars) and F2 sham (purple bars) pups during the P1-4 and P5-10 periods. * p < 0.05. For each group we used the following number of pups: F1 sham, n = 55, females = 31, males = 24 ; F2 sham, n = 24, females = 12, males = 12 (PNG 105 KB)

Figure S5: Weight and length of F2 sham pups. A, Scatter plot illustrating the evolution of body weight of sham (dark grey) and sham_F2 (purple) pups over the P0-10 period. B, C, Same representation as in A, for females (B) and males (C). D, Bar chart illustrating pups’ weight gain during the P0-1 and P0-10 periods. E-G, Same representation as in A-C of pups’ growth curves. H, Bar chart illustrating pups’ length gain during the P0-1 and P0-7 periods. * p < 0.05. For each group we used the following number of pups: F1 sham, n = 55, females = 31, males = 24 ; F2 sham, n = 24, females = 12, males = 12 (PNG 258 KB)

Figure S6: Weight and Length of Re-Exposed Pups. A, Scatter plot illustrating the evolution of body weight over the P0-10 period of F1 TiO2_200 (blue bars) and F2 TiO2_200 (dark blue bars) pups. B, C, Same representation as in A, for females (B) and males (C). D, Bar chart illustrating pups' weight gain during the P0-1 and P0-10 periods. E-G, Same representation as in A-C of pups' growth curve. H, Bar chart illustrating pups' length gain during the P0-1 and P0-7 periods. * p < 0.05. For each group we used the following number of pups: F1 TiO2_200, n = 36, females = 19, males = 17 ; F2 TiO2_200, n = 24, females = 13, males = 11 (PNG 264 KB)

Figure S7: Apneas Temporal Structure is not aggravated by TIO2NPs Re-Exposure in F2 Pups. A-C, Bar charts illustrating the apnea number (A), the apnea mean duration (B), and total time spent in apnea (C) of F1 TiO2_200 (blue bars) and F2 TiO2_200 (dark blue bars) pups during the P1-4 and P5-10 periods. For each group we used the following number of pups: F1 TiO2_200, n = 36, females = 19, males = 17 ; F2 TiO2_200, n = 24, females = 13, males = 11 (PNG 124 KB)

## Data Availability

The datasets used and/or analysed during the current study are available from the corresponding author on reasonable request.
