# Supplementary figures and images for: Multigenerational inheritance of breathing deficits following perinatal exposure to titanium dioxide nanoparticles in the offspring of mice
Source: Discov Nano. 2024 Jan 23;19(1):16. doi: 10.1186/s11671-023-03927-0 (PMC10805760; doi:10.1186/s11671-023-03927-0)

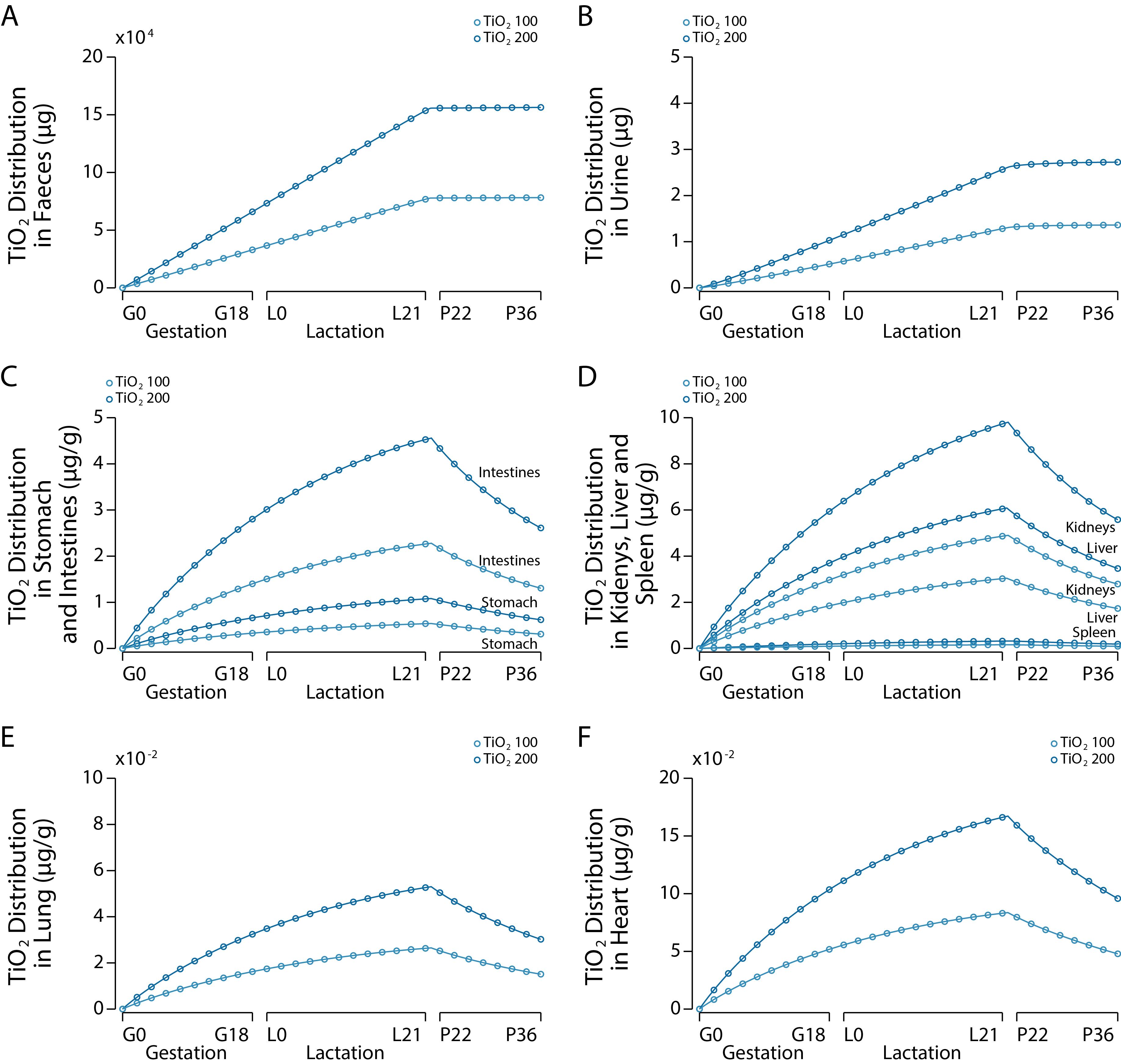

Supplement: Supplementary file 1 — Figure S1: Evolution of tissue concentration of TIO2NPs in exposed dams over gestation and lactation periods. Evolution of TIO2NPs concentration in exposed dams in faeces (A), urine (B), stomach and intestines (C), kidneys, liver, and spleen (D), lung (E), and heart (F), for mice exposed to TIO2NPs at 100 mg/kg (light blue dots) and 200 mg/kg (blue dots) during gestation and lactation periods (PNG 542 KB) [file 11671_2023_3927_MOESM1_ESM.png]

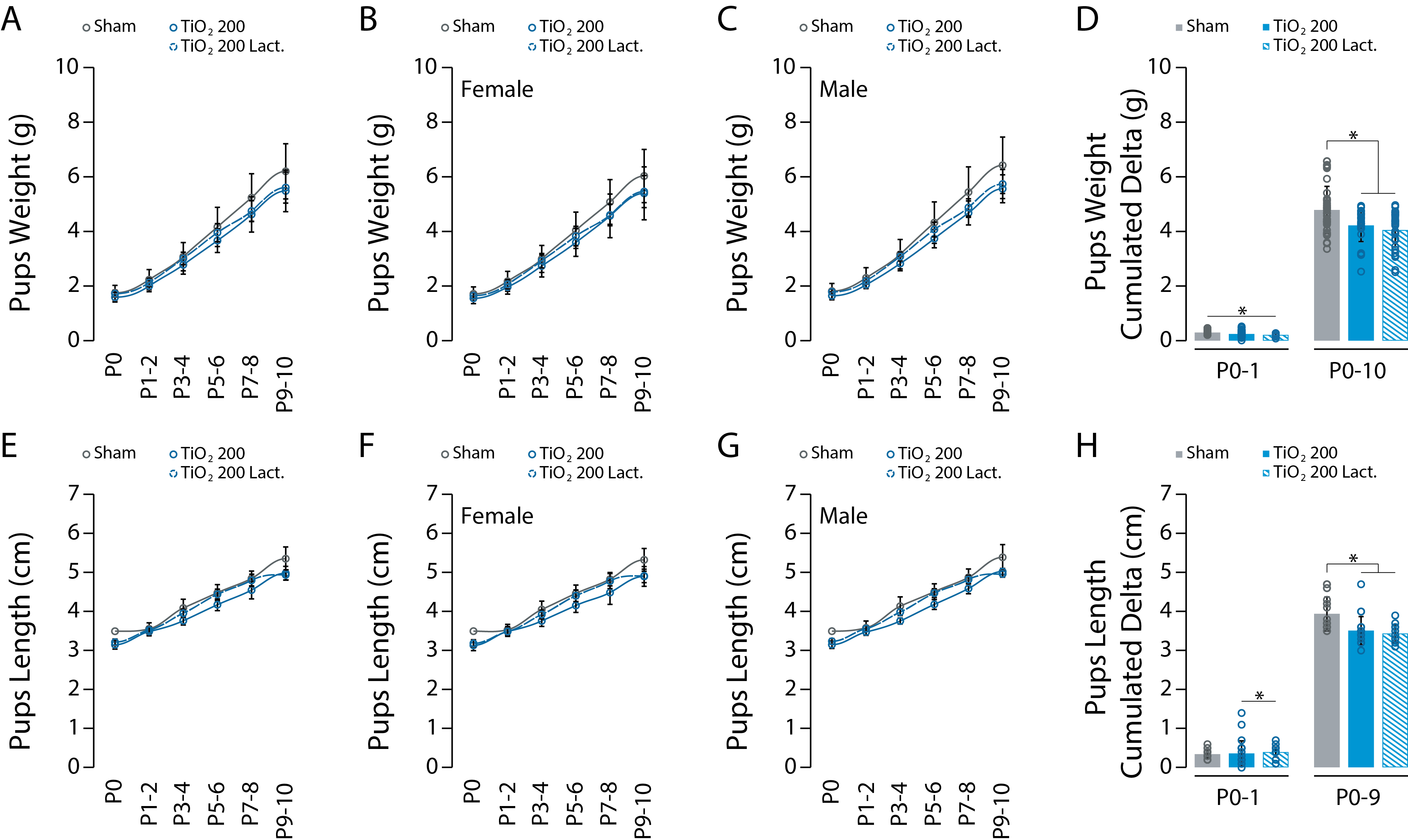

Supplement: Supplementary file 2 — Figure S2: Evolution of pups’ weight and body length following TIO2NPs exposure during lactation. A, Scatter plot illustrating the evolution of body weight of sham (dark grey), TiO2_200 (blue), and TiO2_200 Lact (hatched blue) pups over the P0-10 period. B, C, Same representation as in A, for females (B) and males (C). D, Bar chart illustrating pups’ weight gain during the P0-1 and P0-10 periods. E-G, Same representation as in A-C of pups’ growth curves. H, Bar chart illustrating pups’ length gain during the P0-1 and P0-9 periods. * p < 0.05. For each group we used the following number of pups: sham, n = 55, females = 31, males = 24 ; TiO2_200, n = 36, females = 19, males = 17 ; TiO2_200_Lact, n = 36, females = 17, males = 19 (PNG 343 KB) [file 11671_2023_3927_MOESM2_ESM.png]

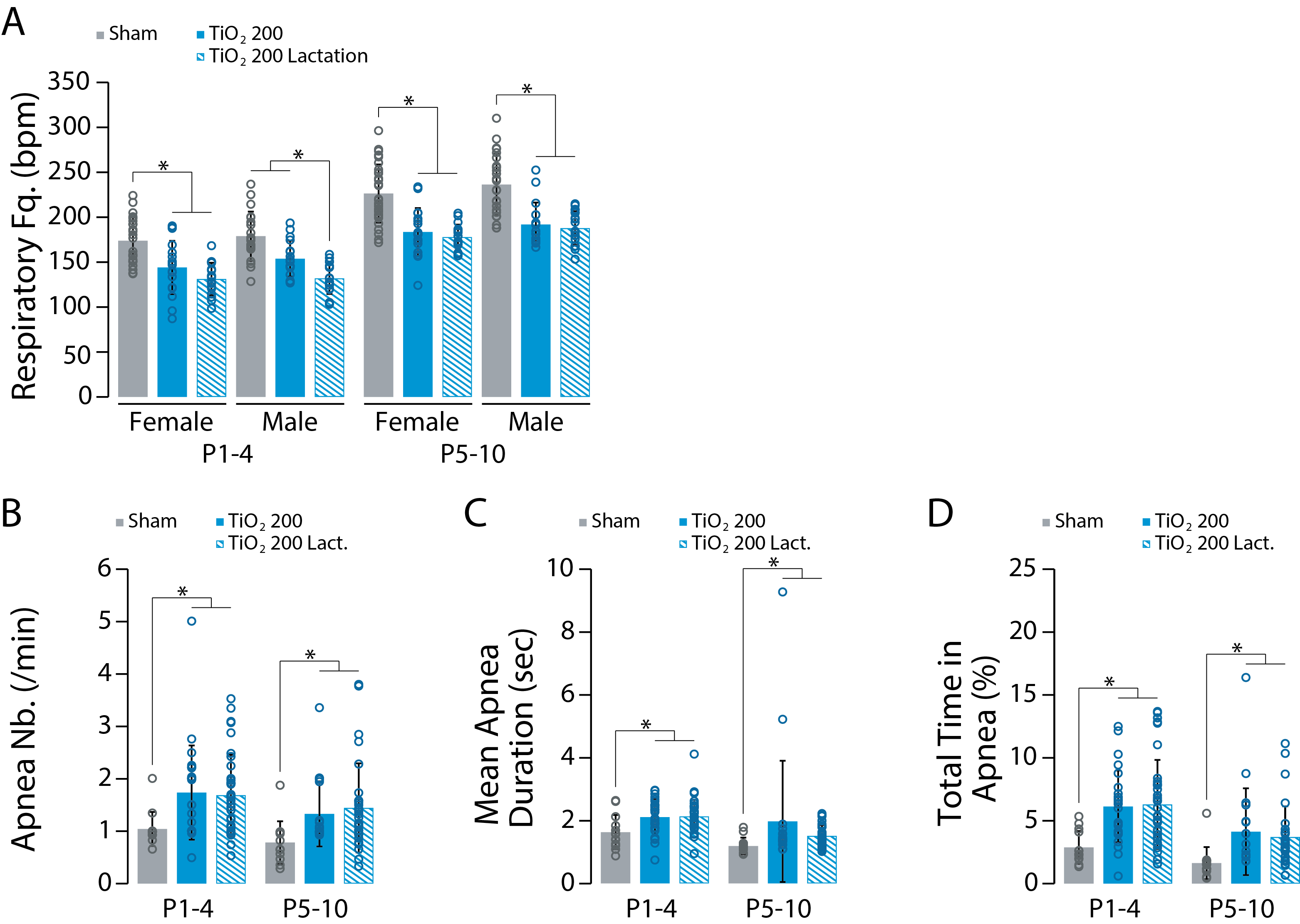

Supplement: Supplementary file 3 — Figure S3: Apneas temporal structure is not affected by TIO2NPs exposure during lactation. A-D, Bar charts illustrating the breathing rate of females and males (A), the number of apneas (B), the mean duration of apneas (C), and the total time spent in apnea (D) of sham (dark grey bars), TiO2_200 (blue bars), and TiO2_200_Lact (blue hatched bars) pups during the P1-4 and P5-10 periods. * p < 0.05. For each group we used the following number of pups: sham, n = 55, females = 31, males = 24 ; TiO2_200, n = 36, females = 19, males = 17 ; TiO2_200_Lact, n = 36, females = 17, males = 19 (PNG 314 KB) [file 11671_2023_3927_MOESM3_ESM.png]

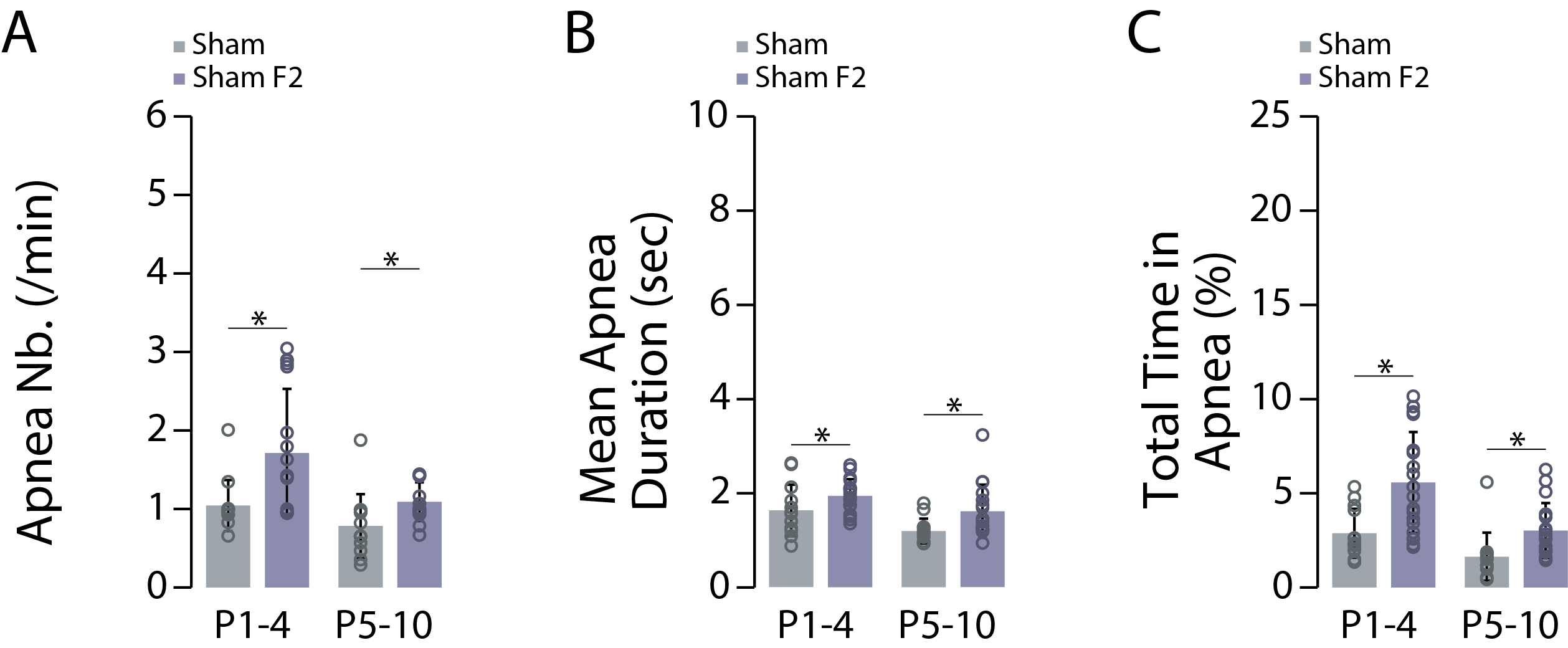

Supplement: Supplementary file 4 — Figure S4: Apneas temporal structure is altered in F2 sham pups. A-C, Bar charts illustrating the number of apneas (A), the mean duration of apneas (B), and the total time spent in apnea (C) of F1 sham (dark grey bars) and F2 sham (purple bars) pups during the P1-4 and P5-10 periods. * p < 0.05. For each group we used the following number of pups: F1 sham, n = 55, females = 31, males = 24 ; F2 sham, n = 24, females = 12, males = 12 (PNG 105 KB) [file 11671_2023_3927_MOESM4_ESM.png]

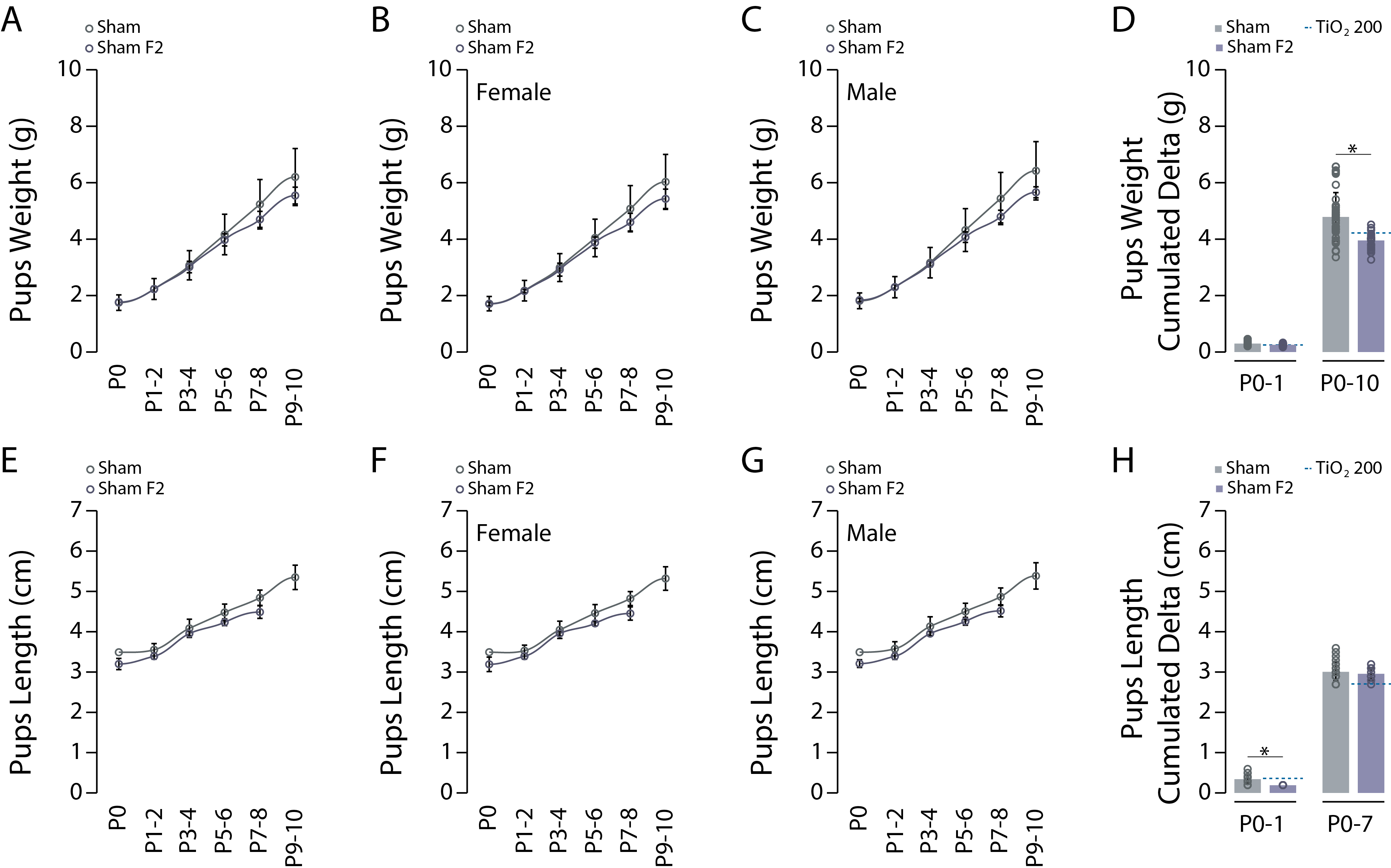

Supplement: Supplementary file 5 — Figure S5: Weight and length of F2 sham pups. A, Scatter plot illustrating the evolution of body weight of sham (dark grey) and sham_F2 (purple) pups over the P0-10 period. B, C, Same representation as in A, for females (B) and males (C). D, Bar chart illustrating pups’ weight gain during the P0-1 and P0-10 periods. E-G, Same representation as in A-C of pups’ growth curves. H, Bar chart illustrating pups’ length gain during the P0-1 and P0-7 periods. * p < 0.05. For each group we used the following number of pups: F1 sham, n = 55, females = 31, males = 24 ; F2 sham, n = 24, females = 12, males = 12 (PNG 258 KB) [file 11671_2023_3927_MOESM5_ESM.png]

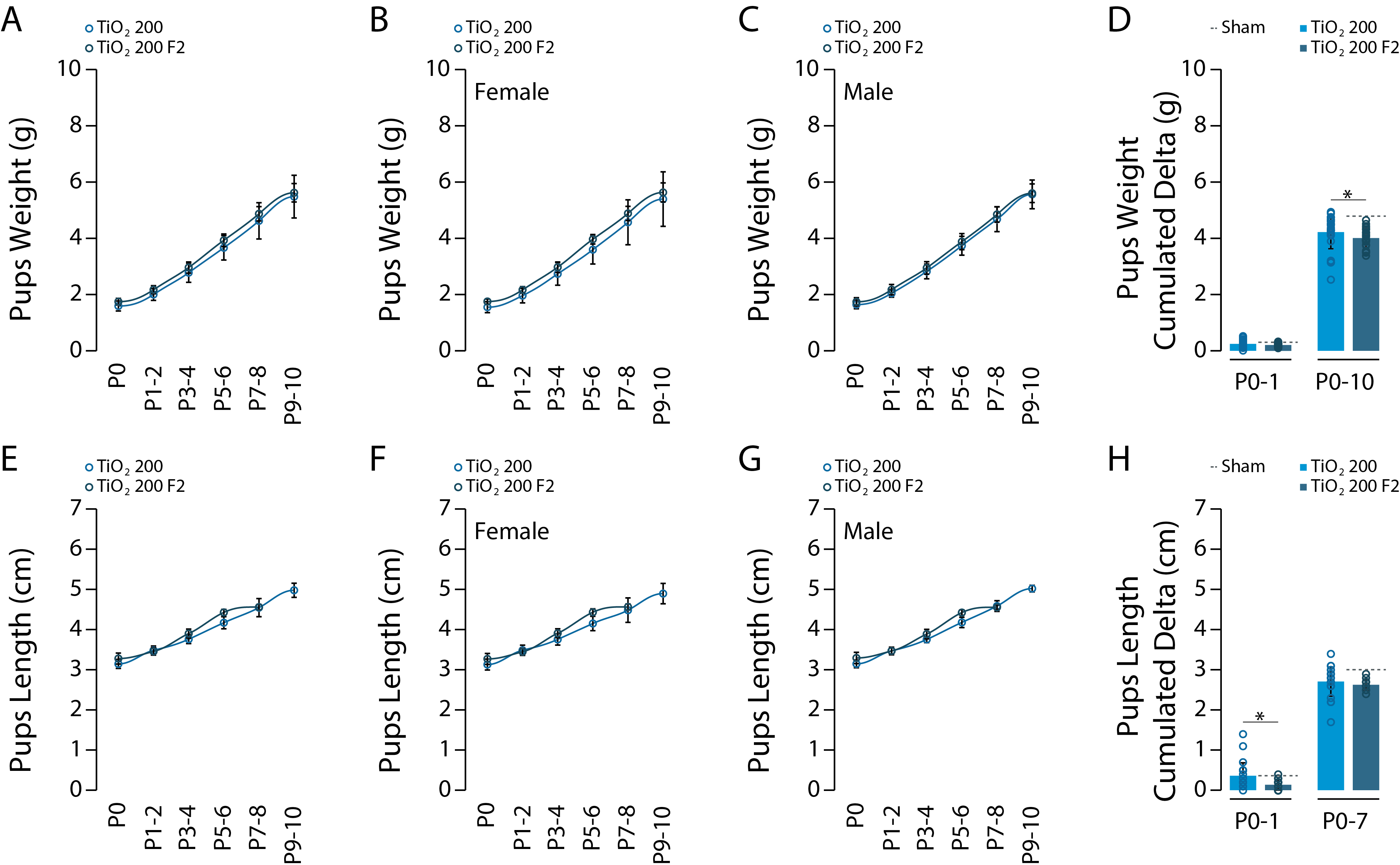

Supplement: Supplementary file 6 — Figure S6: Weight and Length of Re-Exposed Pups. A, Scatter plot illustrating the evolution of body weight over the P0-10 period of F1 TiO2_200 (blue bars) and F2 TiO2_200 (dark blue bars) pups. B, C, Same representation as in A, for females (B) and males (C). D, Bar chart illustrating pups' weight gain during the P0-1 and P0-10 periods. E-G, Same representation as in A-C of pups' growth curve. H, Bar chart illustrating pups' length gain during the P0-1 and P0-7 periods. * p < 0.05. For each group we used the following number of pups: F1 TiO2_200, n = 36, females = 19, males = 17 ; F2 TiO2_200, n = 24, females = 13, males = 11 (PNG 264 KB) [file 11671_2023_3927_MOESM6_ESM.png]

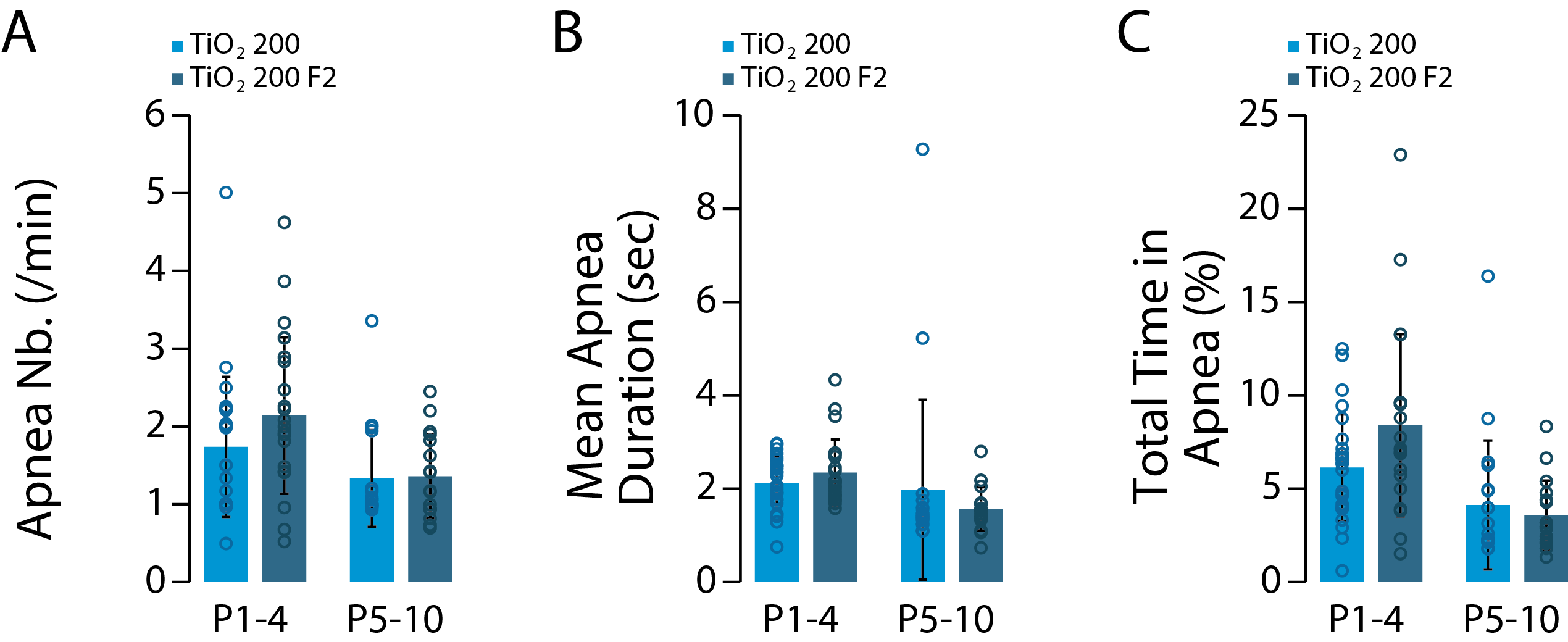

Supplement: Supplementary file 7 — Figure S7: Apneas Temporal Structure is not aggravated by TIO2NPs Re-Exposure in F2 Pups. A-C, Bar charts illustrating the apnea number (A), the apnea mean duration (B), and total time spent in apnea (C) of F1 TiO2_200 (blue bars) and F2 TiO2_200 (dark blue bars) pups during the P1-4 and P5-10 periods. For each group we used the following number of pups: F1 TiO2_200, n = 36, females = 19, males = 17 ; F2 TiO2_200, n = 24, females = 13, males = 11 (PNG 124 KB) [file 11671_2023_3927_MOESM7_ESM.png]
